# Dosimetric Comparison of the Simultaneous Integrated Boost in Whole-Breast Irradiation after Breast-Conserving Surgery: IMRT, IMRT plus an Electron Boost and VMAT

**DOI:** 10.1371/journal.pone.0120811

**Published:** 2015-03-17

**Authors:** Sangang Wu, Youqun Lai, Zhenyu He, Yuan Zhou, Shanyu Chen, Mingming Dai, Juan Zhou, Qin Lin, Feng Chi

**Affiliations:** 1 Xiamen Cancer Center, Department of Radiation Oncology, the First Affiliated Hospital of Xiamen University, Xiamen, People’s Republic of China; 2 Sun Yat-sen University Cancer Center, State Key Laboratory of Oncology in South China, Department of Radiation Oncology, Collaborative Innovation Center of Cancer Medicine, Guangzhou, People’s Republic of China; 3 Xiamen Cancer Center, Department of Obstetrics and Gynecology, the First Affiliated Hospital of Xiamen University, Xiamen, People’s Republic of China; University of Nebraska Medical Center, UNITED STATES

## Abstract

**Objectives:**

To compare the target volume coverage and doses to organs at risks (OARs) using three techniques that simultaneous integrated boost (SIB) in whole-breast irradiation (WBI) after breast-conserving surgery, including intensity-modulated radiation therapy (IMRT), IMRT plus an electron boost (IMRT-EB), and volumetric-modulated arc therapy (VMAT).

**Methods:**

A total of 10 patients with early-stage left-sided breast cancer after breast-conserving surgery were included in this study. IMRT, IMRT-EB and VMAT plans were generated for each patient.

**Results:**

The conformity index (CI) of the planning target volumes evaluation (PTV-Eval) of VMAT was significantly superior to those of IMRT and IMRT-EB (*P* < 0.05). The CI of the PTV Eval-boost of VMAT was better than that of IMRT (*P* = 0.018) and IMRT-EB (*P* < 0.001), while the CI of the PTV Eval-boost of IMRT was better than that of IMRT-EB (*P* = 0.002). The V5, V10 and Dmean in ipsilateral lung with VMAT were significantly higher than IMRT (*P* < 0.05) and IMRT-EB (*P* < 0.05). The Dmean, V5 and V10 in heart with VMAT were significantly greater than those of IMRT and IMRT-EB (*P* < 0.05). There was no significant difference in the OARs between IMRT and IMRT-EB (*P* > 0.05).

**Conclusions:**

Considered the target volume coverage and radiation dose delivered to the OARs (especially the heart and lung), IMRT may be more suitable for the SIB in WBI than IMRT-EB and VMAT. Additional clinical studies with a larger sample size will be needed to assess the long-term feasibility and efficacy of SIB using different radiotherapy techniques.

## Introduction

Whole-breast irradiation (WBI) has become an essential part of the multimodal breast-conserving treatment for breast cancer [1]. Additional tumor bed boost after WBI can further reduce the local recurrence rate in some patients at high risk of breast cancer [2–4]. However, studies have found that the tumor bed boost would affect the cosmetic results [5, 6], thereby altering the effectiveness of breast-conserving therapy. The long-term follow-up results of the 'boost versus no boost' trial conducted by the European Organisation for Research and Treatment of Cancer (EORTC) also found that tumor bed boost and use of photon beam boost were important factors affecting cosmetic results in patients [6]. But it was not the primary goal of this trial to investigate different outcome with different boost techniques [6]. In the last decades, a lot of attention has gone to the development of new techniques to reduce side effects. In case of breast irradiation this means late side effects on skin, heart and lungs.

The standard radiation therapy after breast-conserving surgery is sequential tumor bed boost for 60–66 Gy after the 5-week WBI for a total dose of 45–50 Gy, and the entire duration is 6–7 weeks [7]. However, extended treatment time is resource-intensive, with resultant psychosocial and economic implications. With the evolution of radiation physics, several techniques to deliver a tumor bed boost dose have become available. With the further understanding of the biology of breast cancer radiotherapy, the radiation dose of the breast cancer radiotherapy has changed, and recently, there have been an increasing number of studies on simultaneous integrated boost (SIB) [8, 9]. The rationale is a localized dose enhancement in the area at highest risk without prolonging treatment duration, thus not only providing improved patient comfort but also exploiting the higher sensitivity of breast tumor cells towards larger single doses, which has long been postulated in the linear quadratic model [10]. Daily SIB of 2.3 Gy can shorten the duration of radiation therapy [10], therefore, the inconvenience to the patient and cost to radiation oncology department is particularly attractive. However, more-advanced techniques are required to increase the uniformity of the radiation dose to improve the cosmetic outcomes and reduce the dose to the organs at risks (OARs) to reduce late radiation toxicities.

Currently, the main non-invasive tumor bed boost technique is photon or electron beam irradiation [4]; however, there is no strict standard for the irradiation technique used for the SIB. Compared with the conventional irradiation technique, intensity-modulated radiation therapy (IMRT) can perform boost in the tumor bed on the basis of daily radiotherapy, thereby shortening the duration of radiation therapy [11]. In the present study, we compared three techniques for the SIB: IMRT, IMRT plus an electron boost (IMRT-EB), and volumetric-modulated arc therapy (VMAT). We evaluated the target coverage and radiation dose delivery to the OARs to identify the most suitable technique for the SIB in the WBI after breast-conserving surgery.

## Materials and Methods

### Patients

This study included 10 patients with early-stage breast cancer who underwent radiation therapy after breast-conserving surgery at the Department of Radiation Oncology, the First Affiliated Hospital of Xiamen University from August 2013 to December 2013. The inclusion criteria were as follows: 1) female patient age of 18 years or older with left side breast cancer, who underwent breast-conserving surgery; 2) diagnosis of invasive cancer was confirmed by pathology; 3) axillary lymph node dissection was performed, or sentinel lymph node biopsy was negative; 4) stage of I or II (TIN0M0, T2N0M0) according to the 2009 7th edition of the American Joint Committee on Cancer (AJCC)/Union for International Cancer Control (UICC) staging system; 5) microscopic margins were negative (> 1cm); 6) complete immunohistochemistry results including estrogen receptor (ER), progesterone receptor (PR), and human epidermal growth factor receptor 2 (Her2); 7) adjuvant chemotherapy or endocrine therapy was performed in accordance with standards and guidelines. The study was approved by the ethics committee of the First Affiliated Hospital of Xiamen University. All patients provided written consent for storage of their medical information in the hospital database and for research use of this information. Patient characteristics are listed in Table 1.

**Table 1:** Clinical characteristics of 10 patients.

Characteristic	Value
Age (y)	
Median	39.5
Range	26–54
Menopausal status (*n*)	
Premenopausal	9
Postmenopausal	1
Tumor stage (*n*)	
T1	7
T2	3
Tumor location (*n*)	
UIQ	2
UOQ	6
LIQ	1
LOQ	1
Pathology type (*n*)	
Invasive ductal carcinoma	8
Mucinous adenocarcinoma	2
ER/PR status (*n*)	
Negative	1
Positive	9
Her-2 (*n*)	
Negative	9
Positive	1
Sentinel nodes sampled (*n*)	
No	7
Yes	3
Axillary lymph node dissection (*n*)	
No	3
Yes	7

*Abbreviations*: UIQ = upper inner quadrant, UOQ = upper outer quadrant, LIQ = lower inner quadrant, LOQ = lower outer quadrant.

### Delineation of the target volume and normal tissue

The delineation of the target volume and normal tissue was finally approved by three experienced radiation oncologist. Clinical target volume (CTV) for the whole breast was delineated according to the recommendations of the International Commission on Radiation Units (ICRU) report 83 [12]. The breast CTV included all visible breast parenchyma. The planning target volume of the breast (PTV-breast) added a 7- mm expansion in all directions around the CTV except for the skin surface, including the set-up margin and accounting for patient movement. The PTV for evaluation (PTV Eval-breast) was limited anteriorly to exclude the region outside of the patient and the first 5 mm of tissue under the skin, and was limited posteriorly to no deeper than the posterior surface of the ribs (to exclude lung).

Delineation of the lumpectomy (gross tumor volume, GTV): The GTV was delineated according to the metal mark placed during the surgery, the residual seroma after surgery, mammary gland interruption and density change on CT, and ultrasound findings of patients in the same fixed position including the distance from the tumor bed, i.e., the tumor cavity or interruption of the mammary gland to certain points on the skin, and the depth, width, and height of the tumor bed.

CTV boost included: GTV + 1 cm, limiting the CTV posteriorly to the anterior surface of the pectoralis major, and anterolaterally to 5 mm from skin, not crossing the midline. In general, the pectoralis and/or serratus anterior muscles are excluded from the lumpectomy CTV unless clinically warranted by the tumor pathology. The PTV boost was added to include a 7-mm expansion in all direction around the CTV boost. The PTV Eval-boost was limited anteriorly to exclude the region outside the patient and the first 5 mm of tissue under the skin, and was limited posteriorly to no deeper than the posterior surface of the ribs (to exclude lung).

Delineation of normal organs: The contralateral breast, lung, and heart were delineated on the CT image of each slice. Table 2 shows the volume of target and normal tissue of the entire study population.

**Table 2:** The volume of target and normal tissue of 10 patients.

Target volume and normal tissue	Volume		
	Mean (cm^3^)	Median (cm^3^)	Range (cm^3^)
PTV-Whole breast	473.10	482.03	256.12–731.02
CTV-Tumor bed	27.18	26.33	9.85–50.36
PTV-Boost	68.44	67.65	30.70–103.00
Ipsilateral lung	1086.00	1056.56	809.78–1499.45
Heart	524.50	547.26	364.1–613.32
Contralateral breast	467.51	450.99	298.02–633.56

CTV, clinical target volume; PTV, planning target volume.

### Radiation treatment planning

**Fig 1:**
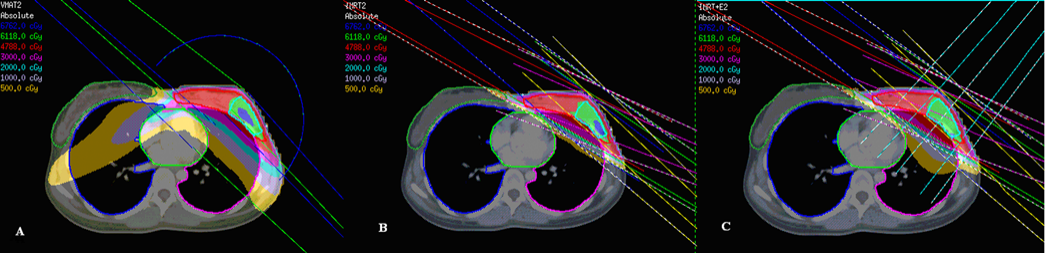
Beam arrangement, target coverage, and doses to normal organs in VMAT (A), IMRT (B), and IMRT-EB (C).

Fig. 1 shows the beam arrangement of the three radiotherapy techniques. In IMRT, 5-field beam with tangential direction were used to deliver a homogeneous dose to PTV-breast and PTV-boost by step and shot IMRT. An angle of 20–30° was used between the two beams in the same direction, and the maximum number of segments was 50. IMRT-EB combined the two programs. The IMRT plan design is described above, but is emphasized only for PTV-breast. IMRT dose prescription was 28 fractions of 1.8 Gy. Electron boost plans used a single electron to each patient's PTV-boost. In order for the PTV-boost to be covered by the 95% isodose line, electron energy of 6, 9, 12 or 15 MeV was chosen according to the depth of the PTV-boost. The doses were then combined according to V95 including the 95% prescribed dose.

In VMAT, double ipsilateral partial arcs with a maximum individual length of 180° starting from the mid-sternum were adopted for this study. Collimator angles were individualized to patients, and ranged from 15° to 30°.

The evaluation criteria for the planned dose for the target area and the dose limitation of the OARs referred mainly to the IMRT-MC2 trial [13]. Radiation treatment of the whole breast included a total dose of 50.4 Gy and 1.8 Gy per fraction with 2.3 Gy per fraction with integrated boost to the tumor bed for a total dose of 64.4 Gy in 28 fractions. With regard to the organs at risk, less than 10% of the heart volume may receive >30 Gy, while less than 20% of the ipsilateral lung may receive >20 Gy. The mean dose to the contralateral breast should be limited to less than 5 Gy. Treatment planning was performed in Pinnacle (Version 9.2, Philips, USA) treatment planning system (TPS). All plans were normalized so that 95% of PTV-breast and PTV-boost received 95% of the prescribed dose (PD).

### Calculation of conformity index (CI) and homogeneity index (HI)

CI = (VTref / VT) × (VTref / Vref), VTref, where VTref represents the target volume covered by isodose; VT is target volume; Vref is the total volume covered by 95% of isodose. CI range was 0–1, in which the conformity was better when the CI value was larger. HI = D5/D95, where D5 represents the irradiation dose received by 5% of PTV-Eval, while D95 represents the irradiation dose received by 95% of PTV-Eval. The closer the HI value is to 1, the better the target uniformity will be.

### Statistical analysis

One-way analysis of variance (ANOVA) test was used to compare dosimetric differences among plans using the three SIB techniques. All statistical tests were two-sided, and were performed using SPSS software (release 17.0, SPSS Inc., Chicago, IL, USA). Statistical significance was defined as *P* < 0.05.

## Results

### Target volume coverage

**Fig 2:**
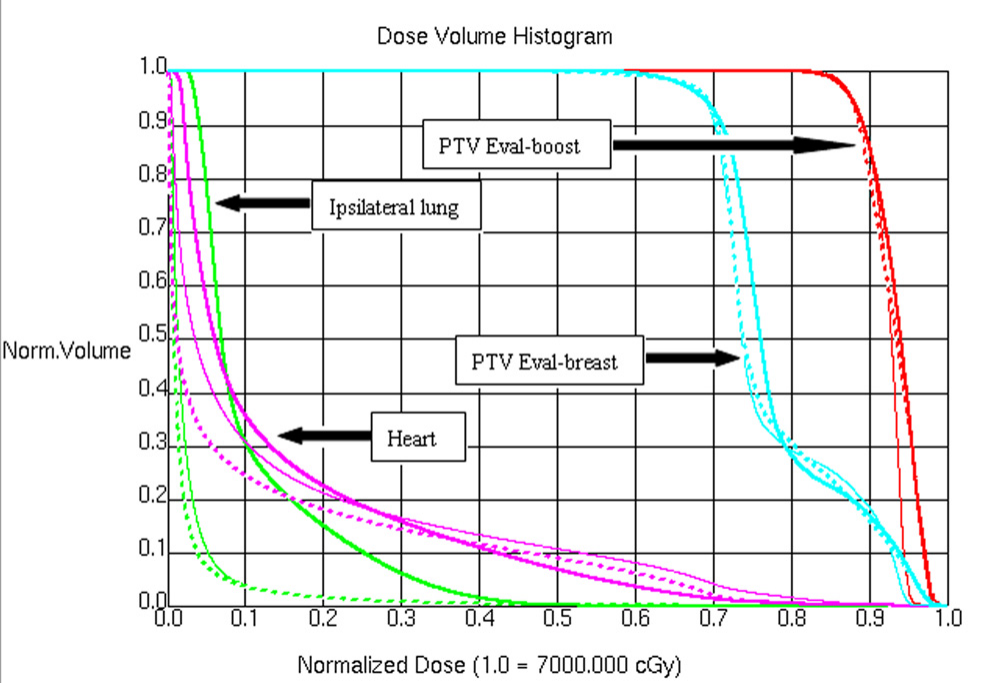
Dose volume histogram of the target volume coverage and normal organs in VMAT (medium solid), IMRT (medium dashed), and IMRT-EB (thin solid).

Table 3 presents dosimetric parameters for IMRT, IMRT-EB and VMAT for the target volume coverage. Figs. 1 and 2 show the target volume coverage and doses to the normal tissue in the three treatment programs.

**Table 3:** Comparison of PTV and normal tissue for IMRT, IMRT plus an electron boost, and VMAT.

Dosimetric parameters	IMRT (A)	FIF-IMRT + electron boost (B)	VMAT (C)	*P*
PTV-WBI				
CI	0.74 ± 0.05	0.75 ± 0.04	0.80 ± 0.05	A vs. B (0.644), A vs. C (0.016), B vs. C (0.045)
HI	1.16 ± 0.04	1.18 ± 0.04	1.13 ± 0.02	A vs. B (0.071), A vs. C (0.038), B vs. C (< 0.001)
V95 (%)	95.57 ± 0.45	95.42 ± 0.33	95.57 ± 0.39	A vs. B (0.407), A vs. C (0.100), B vs. C (0.407)
V105 (%)	14.17 ± 5.13	13.90 ± 6.52	11.43 ± 3.29	A vs. B (0.908), A vs. C (0.245), B vs. C (0.293)
Dmean (cGy)	5073.70 ± 41.72	5084.70 ± 46.62	5070.00 ± 41.64	A vs. B (0.575), A vs. C (0.850), B vs. C (0.455)
Dmax (cGy)	6226.10 ± 131.36	6439.50 ± 145.43	6016.50 ± 90.92	A vs. B (< 0.001), A vs. C (< 0.001), B vs. C (< 0.001)
PTV-boost				
CI	0.84 ± 0.08	0.76 ± 0.05	0.91 ± 0.02	A vs. B (0.002), A vs. C (0.018), B vs. C (< 0.001)
HI	1.07 ± 0.01	1.07 ± 0.01	1.07 ± 0.01	A vs. B (0.583), A vs. C (0.623), B vs. C (0.954)
V95 (%)	95.69 ± 0.40	95.54 ± 0.56	95.82 ± 0.32	A vs. B (0.453), A vs. C (0.515), B vs. C (0.167)
V105 (%)	2.19 ± 2.36	1.38 ± 1.39	1.00 ± 1.27	A vs. B (0.308), A vs. C (0.141), B vs. C (0.635)
Dmean (cGy)	6401.70 ± 55.40	6426.60 ± 51.95	6444.20 ± 18.44	A vs. B (0.287), A vs. C (0.061), B vs. C (0.391)
Dmax (cGy)	6723.10 ± 81.70	6737.70 ± 102.01	6776.10 ± 96.63	A vs. B (0.731), A vs. C (0.273), B vs. C (0.154)
Ipsilateral lung				
V5(%)	18.09 ± 6.72	19.26 ± 7.45	39.78 ± 3.06	A vs. B (0.670), A vs. C (< 0.001), B vs. C (< 0.001)
V10(%)	12.65 ± 4.91	12.74 ± 4.98	24.86 ± 2.74	A vs. B (0.961), A vs. C (< 0.001), B vs. C (< 0.001)
V20(%)	9.24 ± 4.19	9.27 ± 4.19	12.12 ± 1.33	A vs. B (0.983), A vs. C (0.077), B vs. C (0.080)
V30(%)	6.96 ± 3.64	7.03 ± 3.67	4.90 ± 1.32	A vs. B (0.960), A vs. C (0.145), B vs. C (0.133)
Dmean (cGy)	497.45 ± 185.54	530.07 ± 191.24	760.05 ± 92.74	A vs. B (0.658), A vs. C (0.001), B vs. C (0.004)
Heart				
V5(%)	18.09 ± 6.72	19.26 ± 7.45	39.78 ± 3.06	A vs. B (0.670), A vs. C (< 0.001), B vs. C (< 0.001)
V10(%)	12.65 ± 4.91	12.74 ± 4.98	24.86 ± 2.74	A vs. B (0.961), A vs. C (< 0.001), B vs. C (< 0.001)
V20(%)	9.24 ± 4.19	9.27 ± 4.19	12.12 ± 1.33	A vs. B (0.983), A vs. C (0.077), B vs. C (0.080)
V30(%)	6.96 ± 3.63	7.02 ±3.67	4.90 ± 1.32	A vs. B (0.960), A vs. C (0.145), B vs. C (0.133)
Dmean (cGy)	497.45 ± 185.54	530.07 ± 191.24	760.05 ± 92.74	A vs. B (0.658), A vs. C (0.001), B vs. C (0.004)
Contralateral breast				
Dmean(cGy)	10.29 ± 4.22	18.25 ± 6.25	115.49 ± 21.47	A vs. B (0.187), A vs. C (< 0.001), B vs. C (< 0.001)
MU	538.40 ± 43.88	522.60 ± 71.16	504.00 ± 83.07	A vs. B (0.608), A vs. C (0.268), B vs. C (0.546)

CTV, clinical target volume; PTV, planning target volume; IMRT, intensity-modulated radiation therapy; VMAT, volumetric-modulated arc therapy; CI, conformity index; HI, homogeneity index (HI); MU, monitor unit.

The CI and HI of the breast PTV-Eval of VMAT were significantly superior to those of IMRT and IMRT-EB (*P* < 0.05), while the CI and HI of the breast PTV-EVAL were not significantly different between the IMRT and IMRT-EB (*P* > 0.05). There was no significant difference in the Dmean, V95, and V105 among the three radiotherapy techniques (*P* > 0.05), but the Dmax of the breast PTV-Eval of VMAT was significantly lower than that of IMRT (*P* < 0.001) and IMRT-EB (*P* < 0.001). The Dmax of the breast PTV-Eval of IMRT was significantly lower than that of the IMRT-EB (*P* < 0.001).

The CI of the breast PTV-Eval-boost among the three radiotherapy techniques were significantly difference. The CI of the breast PTV-Eval-boost of VMAT was better than that of IMRT (*P* = 0.018) and IMRT-EB (*P* < 0.001), and the CI of the PTV-Eval-boost of IMRT was better than that of IMRT-EB (*P* = 0.002); however, there was no significant difference in the HI, Dmean, Dmax, V95 and V105 among the three radiotherapy techniques (*P* > 0.05).

### Doses to normal organs

Table 3 presents the results of dosimetric comparison of ipsilateral lung and heart for 10 patients with left-sided breast cancer. The V5 and V10 of ipsilateral lung in VMAT was significantly higher than that of IMRT (*P* < 0.05) and IMRT-EB (*P* < 0.05), and there was no significant difference in the V5 and V10 between IMRT and IMRT-EB (*P* = 0.670).

There was no significant difference in the V20 and V30 of ipsilateral lung among the three radiotherapy techniques (*P* > 0.05) (Figs. 1 and 2). There was no significant difference in V5, V10, V20, and V30 of ipsilateral lung between IMRT and IMRT-EB (*P* > 0.05). The Dmean of ipsilateral lung in VMAT was significantly higher than that of IMRT (*P* = 0.001) and IMRT-EB (*P* = 0.004), and there was no significant difference between IMRT and IMRT-EB (*P* = 0.658).

The V5 and V10 of heart in VMAT were significantly higher than that of IMRT and IMRT-EB (*P* < 0.05), and there was no significant difference in the V5 or V10 between IMRT and IMRT-EB (*P* > 0.05). The V20 and V30 of heart in VMAT were not significantly different from those of IMRT and IMRT-EB (*P* > 0.05). The Dmean of heart in IMRT and IMRT-EB was significantly less than that of VMAT (*P* < 0.05). The V5, V10, V20, V30 and Dmean were not significant difference between IMRT and IMRT-EB (*P* > 0.05).

The Dmean of the contralateral breast in VMAT were significantly higher than that of IMRT and IMRT-EB (*P* < 0.05), and there was no significant difference between IMRT and IMRT-EB (*P* = 0.187). There was no significant difference in the MU among the three radiotherapy techniques (*P* > 0.05).

## Discussion

In the present study, we compared the target volume coverage and dose to the OARs of IMRT, IMRT-EB, and VMAT, the three techniques that SIB in WBI after breast-conserving surgery. The results showed that although VMAT had better target volume coverage, its extent of low-dose irradiation of the heart and lung was significantly greater than that of the other two techniques. The target volume coverage of IMRT was slightly lower than that of VMAT, but it was superior to that of IMRT-EB.

The CI and HI of target volume coverage are important indicators for the assessment of the radiotherapy techniques, and they might be important factors affecting the cosmetic effects of breast-conserving therapy. Early findings of EORTC 'boost versus no boost' randomized trial showed that tumor bed boost performed using the electron beam, photon beam, and interpolation technique did not cause significantly different cosmetic effects [14]; however, longer follow-up found that the photon beam irradiation was an important factor affecting the cosmetic results [6]. A possible reason may be related to the dosimetric characteristics of the photon beam itself and the fact that radiotherapy technique was underdeveloped at that time. Thus, more volume of normal breast tissue underwent high-dose irradiation, thereby affecting the cosmetic results. The following dosimetric studies on the photon beam and electron beam boost found results opposite to those of the CI of the photon beam [15, 16]. Currently, the electron beam is a widely adopted tumor bed boost technique because its dosimetric characteristics are more suitable for superficial tumor bed; however, boost for deeper tumor bed requires increased electron beam energy, which will further increase the radiation dose to the heart, lung and normal breast tissue. There has been no standard for the SIB in WBI after breast conserving surgery so far. Therefore, it is particularly important to explore a more precise conformal irradiation mode that is appropriate for the contemporary treatment program.

The dosimetric characteristics of IMRT and VMRT make radiotherapy with SIB feasible. At present, there are no studies on the integrated tumor bed boost techniques, IMRT, IMRT-EB, and VMAT in WBI after breast conserving surgery. In study on WBI, Jin et al. found that the CI of the VMAT target volume was lower than that of tangential field IMRT (*P* < 0.05), and the HI of the target volume was even worse (*P* < 0.05). Therefore, VMAT was not recommended for left-breast cancer in patients with small breasts [17]. In another study on the sequential tumor bed boost after WBI, it was found that the Arc technique achieved a better CI, but the doses to the heart, ipsilateral lung and contralateral breast were higher than those delivered by other irradiation modes [15]. In addition, the range of the dose build-up region was wider. The target volume V95% of the two-field photon beam boost mode was better than that of the electron beam (94.44% vs. 79.91%), but its CI of target volume was poorer than that of the electron beam (39 vs. 47) [15]. Park et al. found that the HI and CI of in target volume of the photon beam boost were superior to those of the electron beam, and the volume of normal organs receiving high doses of radiation was reduced, accompanied by an increase in the volume of normal organs receiving low doses of radiation [16].

In our study, the CI of target volume with VMAT was better than that of IMRT and IMRT-EB, and the CI of the target volume with IMRT was better than that of IMRT-EB; however, there was no significant difference in the HI among the three techniques. Moreover, the range of the ipsilateral lung and heart receiving low doses of radiation in VMAT was greater than that in IMRT and IMRT-EB. The clinical results of SIB in whole-breast irradiation with different techniques is still lacking. The radiotherapy planning is based on CT images in modern age, once the target volume coverage meets the requirements that will not affect local control. Therefore, the technique that can minimize the doses to OARs has become the most appropriate choice. In present study, we found that IMRT is superior to the other two techniques when achieving comparable target volume coverage and doses to OARs, and it is a more appropriate method for the SIB.

Target volume movement due to respiration-induced organ motion is an important factor affecting the radiation dose received by target volume and OARs during breast IMRT. Zhang et al. has developed a model to estimate 3-dimensional motion in patients CT images for predicting respiration-induced organ motion that has potential for improving the accuracy of dose calculation in radiotherapy [18]. In addition, the active breathing control (ABC) technique can reduce or eliminate target volume caused by respiration, and more importantly, the ABC technique can decrease the radiation volume and dose to the lung and heart to reduce radiation injury. Remouchamps et al. reported that deep tangential field whole-breast IMRT was applied together with the ABC technique and the dose distribution in the IMRT target volume coverage became more homogeneous compared with conventional tangential field irradiation, and the radiation dose to the heart and lung was significantly reduced in the moderate deep inspiration breath hold compared with the free breathe [19].

With the improved efficacy of breast cancer therapy, the likelihood of long-term survival of patients may be increased. Therefore, radiation toxicities during long-term follow-up are the subject of increasing attention. Henson et al. studied 558,871 patients with breast cancer who underwent postoperative radiotherapy, and they found that the 30-year radiotherapy-related mortality of lung cancer was higher than radiotherapy-related mortality during the first 20 years after radiotherapy (*P* = 0.002) [20]. VMAT may lead to more scattered radiation to the surrounding normal organs due to its own technical characteristics [21], especially to lung tissue. Dosimetric studies have shown that tangential field IMRT could markedly reduce the risk of lung cancer compared with multi-direction IMRT and VMAT [22]. The ipsilateral lung V5 and V10 of VMAT was significantly higher than that of IMRT and IMRT-EB, indicating that VMAT is associated with a higher possibility of radiation-induced lung cancer, but the results of long-term follow-up are still lacking.

The survival advantage after radiotherapy for breast cancer was probably offset by increased cardiovascular mortality in early series because of inferior radiotherapy techniques and choice of target volume such as internal mammary lymph nodes. Darby et al. analyzed patients treated for breast cancer between 1958 and 2001 and found that the probability of ischemic heart disease started to increase after several years post radiotherapy, and such increase would last for at least 20 years [23]. Another study conducted long-term follow-up in 35,000 patients undergoing radiotherapy, and found that the incidence of cardiovascular disease increased remarkably after radiotherapy [24]. One study pointed out that cardiovascular mortality was not increased in patients who underwent radiotherapy after 1980 [25], but the effects of radiation therapy on the cardiovascular system will become apparent only after a long period of time, perhaps 30–40 years [20, 26]. This study showed that the region of the heart receiving low doses of radiation in VMAT was greater than that in IMRT and IMRT-EB, and there has been no report on the long-term follow-up results of VMAT for the treatment of breast cancer [27]. Therefore, further studies are warranted to explore whether VMAT is suitable for the clinical treatment of patients with breast cancer.

## Conclusions

In conclusion, our study results suggest that IMRT may be more suitable for the simultaneously integrated tumor bed boost in WBI after breast-conserving surgery than IMRT-EB and VMAT when ensuring appropriate target coverage and considering doses to OARs, especially the heart and lung. However, since the conclusion of the present study is based on a dosimetry study and the sample size is small. Additional clinical studies with a larger sample size will be needed to assess the long-term feasibility and efficacy of SIB using different radiotherapy techniques.

## Supporting Information

S1 Dataset The data underlying the findings in present study. (XLS)
